# Acoustic signalling and behaviour of Antarctic minke whales (*Balaenoptera bonaerensis*)

**DOI:** 10.1098/rsos.211557

**Published:** 2022-07-27

**Authors:** C. B. Casey, S. Weindorf, E. Levy, J. M. J. Linsky, D. E. Cade, J. A. Goldbogen, D. P. Nowacek, A. S. Friedlaender

**Affiliations:** ^1^ Institute for Marine Sciences, Long Marine Laboratory, University of California Santa Cruz, 115 McAllister Way, Santa Cruz, CA 95060, USA; ^2^ Ocean Sciences Department, University of California Santa Cruz, 115 McAllister Way, Santa Cruz, CA 95060, USA; ^3^ Department of Biology, Hopkins Marine Station, Stanford University, 120 Ocean View Blvd, Pacific Grove, CA 93950, USA; ^4^ Nicholas School of the Environment and Pratt School of Engineering, Duke University Marine Laboratory, 135 Duke Marine Lab Road, Beaufort, NC 28516, USA

**Keywords:** acoustic communication, baleen whales, biologging, Antarctic minke whales, behaviour

## Abstract

Acoustic signalling is the predominant form of communication among cetaceans. Understanding the behavioural state of calling individuals can provide insights into the specific function of sound production; in turn, this information can aid the evaluation of passive monitoring datasets to estimate species presence, density, and behaviour. Antarctic minke whales are the most numerous baleen whale species in the Southern Ocean. However, our knowledge of their vocal behaviour is limited. Using, to our knowledge, the first animal-borne audio-video documentation of underwater behaviour in this species, we characterize Antarctic minke whale sound production and evaluate the association between acoustic behaviour, foraging behaviour, diel patterns and the presence of close conspecifics. In addition to the previously described *downsweep* call, we find evidence of three novel calls not previously described in their vocal repertoire. Overall, these signals displayed peak frequencies between 90 and 175 Hz and ranged from 0.2 to 0.8 s on average (90% duration). Additionally, each of the four call types was associated with measured behavioural and environmental parameters. Our results represent a significant advancement in understanding of the life history of this species and improve our capacity to acoustically monitor minke whales in a rapidly changing Antarctic region.

## 1. Introduction

The field of bioacoustics aims to describe the breadth of sounds animals produce and elucidate their function. To accomplish this, detailed acoustic recordings must first be paired with observations of animal behaviour. Subsequently, carefully designed experiments can then test the significance of a given sound type to its intended listener. This approach has provided a wealth of new knowledge for terrestrial animals; however, accurately pairing sound production with social and environmental variables remains difficult for animals that live in aquatic habitats.

Despite baleen whales having evolved to use sound as their primary means of communication under water, relatively little is known about the behavioural significance of their acoustic signals. This is because animals are only observable for short periods at the water's surface, and the context of call production (especially in the presence of conspecifics) is difficult to document. Passive acoustic monitoring systems remain the most effective method for collecting large amounts of acoustic data; however, recordings often lack associated information about the age, sex, behavioural state, and—in many cases—even species of the detected animals.

Multiple methods have been developed to evaluate the behavioural context of baleen whale acoustic behaviour. These include the coupling of underwater recordings with visual observations of animal behaviour at and just below the water's surface [1–4] and the development of biologging tags that sample both acoustic and fine-scale physical behaviour of individual whales (e.g. [5–8]). Despite these advances, the behavioural context associated with sound production has been determined for only a few species including humpback whales (*Megaptera novaeangliae)*, southern and North Atlantic right whales (*Eubalaena australis and glacialis)*, blue whales (*Balaenoptera musculus)* and fin whales (*Balaenoptera physalus)* [3,5,7–15]. Humpback whales produce elaborate songs associated with breeding [13,16,17], ‘feeding calls’ during group foraging on herring in Alaska [10,18,19], and unique clicking [8] and repetitive burst sounds during night-time foraging at depth [11]. Among North Atlantic right whales, the ‘gunshot’ sound is produced by adult males both alone and in group settings at the surface. This vocalization probably functions as an advertisement signal to females and an agonistic signal directed towards other males [12]. Studies of fin whales have revealed that males produce calls or song [20], and that these calls are more frequent during shallow and long-duration dives and vocal behaviour is associated with travelling in groups [21]. Among blue whales, there appear to be clear patterns of behaviour, sex, and group size among different call types [5].

While these studies have been beneficial to assessing vocal production and its relationship to some components of behaviour, many do not provide specific data on the surrounding social or environmental parameters that may influence sound production (e.g. the presence of conspecifics, prey, or environmental features). A novel advancement for overcoming this limitation is integrating video cameras into acoustic and motion-sensing tags. Such bio-loggers provide information on kinematic signatures associated with behaviours such as feeding [22,23], can measure the environment as experienced by the tagged animal through video (e.g. [24]), and document the visibility and relative abundance of other animals within the camera's field of view. Thus, combining motion, video and audio data streams presents a powerful new sampling tool to understand the relationships between behaviour, environmental context and vocal production.

Antarctic minke whales (*Balaenoptera bonaerensis*) are among the most abundant—yet poorly understood—cetacean species owinge to the logistical difficulties of working in their preferred habitat of polar sea ice [25]. Detailed information on the species' vocal behaviour has come from a single acoustic tagging effort [26] that described a pulsating call referred to as a ‘bio-duck,’ as well as a low-frequency signal referred to as a ‘downsweep’ [26,27]. At a broader scale, passive acoustic recording systems have described the distribution and habitat use of Antarctic minke whales via the presence of these known call types [28–31], with acoustic activity being highly seasonal and strongly associated with the austral winter [29]. No study to date has associated behavioural or environmental context with sound production in this species.

Our work uses a unique dataset from video-recording acoustic and motion-sensing tags to (i) describe Antarctic minke whale vocalizations, and (ii) provide a detailed assessment of the associated fine-scale behavioural and environmental context surrounding sound production. Specifically, we characterize different call types and evaluate their association with foraging behaviour, the presence of conspecifics, and diel patterns. This extensive analysis of Antarctic minke whale vocal production provides foundational knowledge necessary to test specific hypotheses for evaluating the function of these specialized signals.

## 2. Material and methods

### 2.1. Field work

We used Customized Animal Tracking Solutions (CATS.is) tags that included the *daily diary package*. This consisted of tri-axial accelerometers that sampled at 400 Hz; magnetometers and gyroscopes at 50 Hz; and pressure, light, temperature and GPS at 10 Hz. Additionally, these tags included an integrated video camera with a resolution of either 1280 × 720 p or 1920 × 1080 p. Cameras were set to record continuously using ambient light while battery was available; when a light sensor detected dark conditions, the cameras were automatically turned off to conserve power. Video recording capacity ranged between 4–7 h per tag. Each tag used in the study was equipped with a calibrated HTI-96-Min hydrophone (High Tech, Inc., Long Beach, MS, USA; −201 dB re: 1V/µPa, frequency response 2 Hz to 30 kHz, 16 or 32 bit depth, 0 or 2 dB gain) that recorded sound continuously at a sampling rate of 48 kHz throughout the tag deployment.

Tags were deployed in February and March of 2018 and 2019 in two bays on the western side of the Antarctic Peninsula, adjacent to the Gerlache Strait (Andvord and Paradise Bays). Tagging was conducted from 4.8 m rigid-hulled inflatable boats using a 6 m carbon fibre hand pole (as in [32]). Focal animals were approached at low speeds (less than 5 mph) from oblique angles and followed until they surfaced, at which time tags were deployed. The tags—which attach via four silicon suction cups—were generally placed on the dorsal surface of the whale between the blowhole and dorsal fin. Efforts were made to align the tag craniocaudally so that the front of the tag and video cameras were facing forward. Tags remained on the whales for up to 48 h; after this time they lost suction, were shed by the whale, and floated to the surface where they were located and retrieved via an Argos satellite transmitter and a VHF transmitter.

A total of 16 tag deployments with associated hydrophone data were used for analysis in this study. Audio data recorded concurrently with video (when light was available) were used to evaluate the social context of sound production. All acoustic data, irrespective of the presence of associated video data, were used for the characterization of call types and the association of call types with diel patterns and foraging behaviour.

### 2.2. Acoustic recordings and analysis

Spectrograms (Hann window; fast Fourier transform (FFT) size 2046; overlap 95%) of all available audio data were generated using Raven Pro (v. 1.5, Center for Conservation Bioacoustics, 2014). Spectrograms were visually and aurally inspected by two individuals with extensive experience evaluating baleen whale acoustic behaviour from tag data. Recordings were then qualitatively compared to published data for Antarctic minke whales [26–31]. Other soniferous species that may have been present in this study area include humpback whales and Antarctic fur seals. The latter species are primarily vocal in air (see [33,34]), and recorded signals were qualitatively compared to published descriptions of Antarctic humpback whale vocalizations from this region [35,36]. Our recordings were then categorized into several descriptive types based on perceptual visual and acoustic similarities (neither the classification structure nor the number of groups was pre-determined). Discrete calls were defined as units of sound that could be readily identified and counted and separated from other signals by more than 3 s of silence. Only the vocalizations belonging to descriptive call types that were present on a minimum of five recordings on at least two separate tag deployments were included in subsequent analyses. A total of 651 suspected minke whale vocalizations were identified and qualitatively assigned to descriptive call categories.

We used signal-to-noise ratios (SNR) to attribute calls to the immediate tagged individual or associated group. A relative SNR of 10 dB was the minimum required to include identified calls in further analysis. Factors including the swimming speed of the animal and the local ambient noise conditions may have led to a conservative selection bias in the signals included. For example, if calls were produced during periods with high background noise and were not salient (e.g. rapid acceleration or surfacing), these would not have met our criteria and been included in further analysis. Given that our objectives were focused on evaluating the broader behavioural and diel contexts associated with sound production, we are confident that the calls used for analysis were either produced by the tagged individual or a close associate in the group. This assumption was also corroborated by limited field observations collected concurrently with tag data.

From the manually audited data, a subsample of high-quality calls for each descriptive call type were selected for additional analysis. Only signals where all parameters of the spectral contour could be identified were included. A total of five acoustic features were measured for each call in Raven Pro (Hann window; low-pass filter 2000 Hz, FFT size 4096; frequency resolution 12 Hz, overlap 95%): duration 90% (s), centre frequency (Hz), first and thrid quartile frequencies (Hz) and 90% bandwidth (Hz). The 90% call duration and bandwidth were selected because they minimize measurement error. Temporal parameters were always measured from the waveform, and spectral parameters were measured from the spectrum or spectrogram. Of all the calls identified, 230 vocalizations were deemed suitable for this detailed acoustic analysis (table 1).

**Table 1.** Comparison of measured call parameters for four different Antarctic minke whale call types (*downsweep, rumble, boom and growl).* (Table includes the total number of high-quality samples (*n*) used for both the acoustic and linear discriminant analyses. Mean values ± standard deviation are provided for first quartile (Q1), centre, third quartile (Q3) and peak frequencies (Hz), the 90% bandwidth (Hz) and the 90% call duration (s). Information on whether the call types were previously described is also provided, with associated references.)

call type	*n*	Q1 frequency (Hz)	centre frequency (Hz)	Q3 frequency (Hz)	90% bandwidth (Hz)	peak frequency (Hz)	90% call duration (s)	previously published
*downsweep*	78	83 ± 17	93 ± 19	105 ± 37	71 ± 37	92 ± 19	0.2 ± 0.1	Schevill & Watkins [27], Risch *et al*. [26], Dominello & Širović [28], Shabangu *et al*. [30]
*rumble*	88	108 ± 53	149 ± 80	286 ± 173	644 ± 256	108 ± 59	0.2 ± 0.1	n.a.
*boom*	26	90 ± 27	102 ± 27	120 ± 35	133 ± 88	96 ± 32	0.8 ± 0.5	n.a.
*growl*	38	133 ± 77	178 ± 97	229 ± 122	244 ± 126	174 ± 111	0.4 ± 0.3	n.a.

We conducted a linear discriminant analysis (LDA) with cross-validation using the five variables measured for each call type to confirm the initial categorization of sounds by trained observers. LDA uses a linear combination of values from two or more independent discriminating variables that best group cases into their *a priori* assigned classes. In this analysis, we assigned call type as the group identifier and acoustic measures as the discriminant variables. Per cent correct classification obtained from the classification matrix (generated by the LDA) provided a metric of how well the measured variables separated the calls into each subjective call category. This analysis provides a relative confidence score for the accuracy of the human-derived call classifications.

### 2.3. Association of call types with behavioural and environmental variables

#### 2.3.1. Diel calling rates

To evaluate whether specific calls were associated with either day or night, we first determined sunrise and sunset times for each tag deployment using the NOAA ESRL sunrise/sunset calculator (https://www.esrl.noaa.gov/gmd/grad/solcalc/sunrise.html). Diel period (day/night/twilight) was determined from the angular sun position at a given location and time using the MATLAB package ‘Sunrise Sunset’ (https://www.mathworks.com/matlabcentral/fileexchange/55509-sunrise-sunset). Twilight was defined as the period when solar elevation was less than 6° below the horizon and was determined in order to exclude those transitional time periods from daylight and night-time hours. All identified calling events were then categorized as being produced either during day or night. To determine diel patterns in calling rates for each call type, we divided the total number of daytime calls by the total number of daylight tag hours. This provided a measure of call rate. We then replicated this process for night-time calls. This allowed us to determine the hourly calling rates for each call type during day and night, by tag deployment. We then compared the proportion of each identified call type's cumulative occurrence during daytime and night-time periods across tag deployments.

#### 2.3.2. Foraging versus non-foraging

To evaluate whether specific calls were associated with either foraging or non-foraging behaviour, we used previously developed methods for evaluating kinematic and motion data to determine the timing of lunge feeding events [37–39]. All kinematic and motion tag data were decimated to 10 Hz, tag orientation on the animal was corrected for, and animal orientation was calculated using custom-written scripts in MATLAB 2014a [6,37–39]. Animal speed for all deployments was determined using the amplitude of tag vibrations [40], and the norm of the jerk signal (vector sum of the difference) was calculated using the ‘njerk’ tool at animaltags.org. Animal speed and jerk were the primary metrics used to characterize the kinematic signature of a rorqual feeding lunge, which includes an increase in speed and overall body acceleration followed by a rapid deceleration [41]. For each recorded dive, we used the presence of one or more lunges to label a dive as feeding. Additionally, we corroborated feeding lunges that occurred during periods when tag video was recorded. We generated a record of foraging and non-foraging dives for each tag deployment, which included both the dive depth and time of day. We compared the proportion of each identified call type occurring during foraging and non-foraging states. The resulting matrix was evaluated using chi-square analysis to determine whether calls were produced more or less frequently among these categories compared to parity.

#### 2.3.3. Presence of close conspecifics

Visual audits were performed on available video data to determine whether close conspecifics were visible or not visible within the video frame during tag deployments. Our analysis of close conspecifics is therefore limited to daylight hours. A total subset of 283 calls occurred concurrently with animal-borne video data. We evaluated all recordings made within a 60 s window of each call (30 s before and 30 s after) to determine if close conspecifics were visible or not within the field of view of the video. This conservative approach limits false positives but may underestimate the presence of conspecifics outside of the view of the camera. Additionally, this approach measures the visibility/non-visibility of close conspecifics surrounding each identified call rather than an estimate of true group size. We then determined the proportion of call types produced when close conspecifics were either visible or not visible and assessed these two categories for biases of occurrence using chi-square analysis.

## 3. Results

A total of 16 tag deployments with associated hydrophones were used in this study. This resulted in a total of 140 h of useable audio data (electronic supplementary material, table S1); of which 81 h and 30 min occurred during the day and 58 h 30 min during the night. From these recordings, a total of 651 suspected minke whale vocalizations were identified and assigned to descriptive call categories. All calls had associated tag sensor/temporal data and were therefore used to evaluate the relationship of different call types with foraging behaviour and diel patterns. A total of 283 calls occurred concurrently with animal-borne video. This subset of data was used to determine whether close conspecifics were visible or not visible during the emission of different call types.

### 3.1. Vocal behaviour of Antarctic minke whales

**Figure 1.**
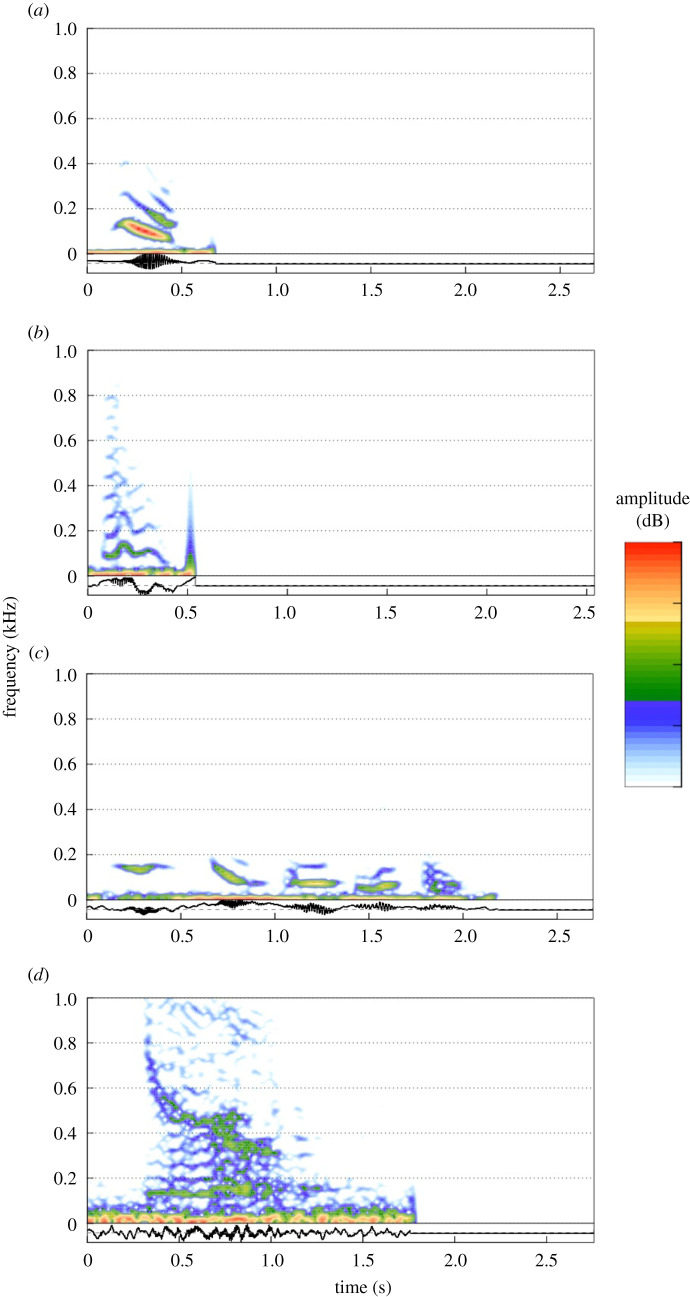
Spectrograms and associated waveforms for the four call types produced by Antarctic minke whales: (*a*) *downsweep*, (*b*) *rumble*, (*c*) *boom,* and (*d*) *growl.* Calls were recorded off the western Antarctic Peninsula in 2018 and 2019. Spectrogram parameters: sampling rate 48 000 Hz, FFT size 512, 3 dB filter bandwidth 135 Hz, 90% overlap.

The visual and acoustic audit of our data revealed the presence of four distinct call types. These include one vocalization reported previously for the Antarctic minke whale (the *downsweep*), and three additional novel calls: the *rumble, boom* and *growl* (figure 1).

The *downsweep* (representing 28% of identified calls, *n* = 185 detected across all deployments) was recorded and described previously [26–28,30]. These continuous, low-frequency signals are short in duration (90% duration approx. 0.2 s) with peak energy occurring around 92 Hz ± 19 Hz (78 calls analysed; figure 1 and table 1). Similar in structure to other low frequency (60–130 Hz) *downsweeps* that have been previously recorded during close encounters with Antarctic minke whales (e.g. [26]), this signal had an average start frequency of approximately 107 Hz, and an end frequency of 60 Hz.

Of the three previously undescribed call types, the most prevalent was the *rumble* (*n* = 276 detected representing 42% of identified calls, 88 calls analysed; figure 1 and table 1). This short vocalization has a 90% duration of approximately 0.2 s ± 0.1 and is similar to the sound produced by a zipper. It has strong harmonics up to around 800 Hz with some frequency modulation present. Of all the calls, this signal has the widest 90% frequency bandwidth of 644 Hz ± 256. The *boom* vocalization (*n* = 124 detected representing 19% of identified calls, 26 calls analysed) is a discrete, guttural, low-frequency signal (average peak frequency approx. 96 Hz ± 32). This signal is often emitted in bouts that last on average 0.8 s (figure 1 and table 1). Finally, the *growl* is a continuous roar with a mean duration of approximately 0.4 s ± 0.3 and a peak frequency that is higher than the other call types of around 174 Hz ± 1121 (*n* = 66 detected representing 10% of calls, 38 calls analysed; figure 1 and table 1).

The LDA accurately differentiated between these four descriptive call types with an overall average agreement of 84%. *Downsweeps* (*n* = 78) were correctly assigned with a classification rate of 98%. *Rumbles* (*n* = 88) were correctly assigned with a classification rate of 90%. *Boom* vocalizations (*n* = 26) were correctly assigned to this call type with a classification rate of 61%. Finally *growls* (*n* = 38) were correctly assigned with a classification rate of 62%. Notably, *growl* vocalizations were often misclassified as *downsweeps* in the LDA classification.

### 3.2. Association of call types with measured environmental and behavioural variables

#### 3.2.1. Diel calling rates

**Figure 2.**
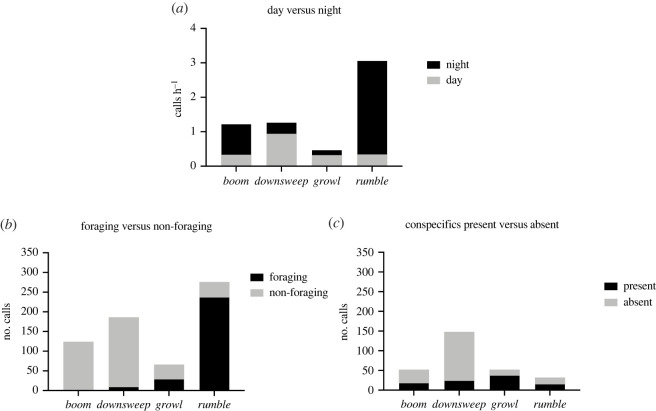
Association of Antarctic minke whale call types with measured environmental and behavioural variables. Calling rates are presented in panel (*a*) for each call type (*n* = 651 calls total) during day and night conditions. Panel (*b*) depicts the proportion of acoustic signals of each type emitted during a foraging versus a non-foraging state (*n* = 651 calls total). Panel (*c*) shows the proportion of calls of each type (*n* = 283 calls total) emitted when conspecifics were present or absent, when concurrent video data was available.

**Figure 3.**
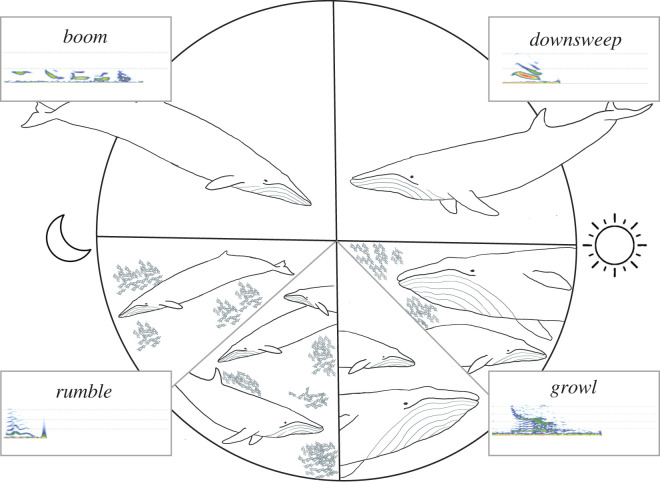
Schematic illustration depicting the association between each call type and specific behavioural and diel conditions. This figure represents the dominant diel condition and foraging state during which each call type was produced. The analysis of whether the call was produced in the presence of close conspecifics is limited to those calls produced during daylight hours when video data was available. *Boom* calls occurred most frequently at night while animals were in a non-foraging state; when they were observed during the day and concurrent video data was available, *booms* were produced when conspecifics were not present. *Rumbles* were similarly emitted most frequently at night and were predominantly made during foraging bouts. When concurrent video data was available, *rumbles* were produced both when conspecifics were present and absent. *Downsweeps* were produced most often during daylight hours and typically occurred in non-foraging contexts when no other whales were within the camera's field of view. *Growls* were also most common during the day and were associated with both foraging and non-foraging behavioural states while animals were in the presence of conspecifics. Line drawing by R. Jones.

All identified call types were strongly associated with either day or night-time conditions (figures 2*a* and 3). *Downsweeps* and *growls* were both more common during daylight hours, whereas *booms* and *rumbles* predominantly occurred at night. *Downsweeps* were 2.8 times more numerous during the day than at night (0.94 calls h^−1^ during the day versus 0.33 calls h^−1^ at night), and *growls* were 2.1 times more prevalent during the day (0.32 calls h^−1^ during the day versus 0.15 calls h^−1^ during the night). Conversely*, booms* were 2.7 times more common at night (0.89 calls h^−1^ at night versus 0.33 calls h^−1^ during the day), and r*umbles* were 8 times more abundant during the night (2.72 calls h^−1^ at night versus 0.34 calls h^−1^ during the day).

#### 3.2.2. Foraging versus Non-foraging

The proportion of calls made during foraging and non-foraging states varied significantly ($\chi_{3,n=651}^{2}=412.9$, *p* < 0.01). Specifically, of all *rumbles* detected across deployments, 86% occurred while animals were in a foraging state (figures 2*b*, 3). Conversely, *downsweeps* and *boom* vocalizations were primarily produced while animals were in a non-foraging state (96% and 100% of calls, respectively). Only the *growl* call type appeared to be emitted frequently during both foraging and non-foraging behavioural states (foraging = 44%, non-foraging = 56%).

#### 3.2.3. Visibility of close conspecifics

The proportion of identified call types emitted in the presence of visible or not visible close conspecifics varied significantly ($\chi_{3,n=283}^{2}=55.85$, *p* < 0.0001). During daylight hours when video data was available, we found that *booms* and *downsweeps* were produced when no close conspecifics were visible within the field of view of the camera (65% and 84%, respectively; figures 2*c* and 3). Conversely, during the same time period *growls* were produced primarily when other minke whales were visible (71% of calls detected). Only *rumbles* were produced consistently both when close conspecifics were both visible and not visible (47% and 53%, respectively).

## 4. Discussion

Through the combined use of acoustic, sensor and video data we have provided, to our knowledge, the first description of vocal production and associated behaviour in the Antarctic minke whale. From a methodological perspective, our study offers a broadly applicable template to enable researchers to investigate the role that calling plays in the daily lives of marine animals. Our results demonstrate that Antarctic minke whales have a larger acoustic repertoire than previously described and that some vocalizations are strongly associated with specific behavioural states and diel patterns. This information provides novel context to both existing and future passive acoustic datasets from the Antarctic and presents a new opportunity to understand the spatio-temporal patterns of behaviour in this species.

Consistent with previous work, the *downsweep* was detected across several deployments. This call type exhibited structural and acoustic similarities to published accounts that used comparable methods in the same geographical region [26]. Our average measured 90% duration for the *downsweep* was 0.2 s—analogous to that reported elsewhere (0.3 s from [28]; 0.2 s from [26])—and most of the energy within the calls from this study fell between 83 and 105 Hz (first quartile and third quartile), which is comparable to other reports for this species (60–130 Hz, [26,28]).

Previous reports noted that *downsweeps* were produced just before or after surfacing while animals were within pack ice [27], and have suggested to serve a social function such as maintaining contact between separated individuals [42,43]. Our results confirm that both the *downsweep* and the *boom* were emitted when no other animals were visible in the camera's field of view and were produced in a non-foraging context. Given that we consistently observed Antarctic minke whales foraging in small groups, we suggest that these calls could function to elicit the attention of other conspecifics in acoustic proximity (i.e. a potential contact call). Contact calls are produced by a wide range of taxa and function to maintain group cohesion and coordinate group movement associated with different behavioural events, including predator avoidance, breeding and group foraging [44].

Several baleen whale species participate in group foraging, leading to increased foraging efficiency (e.g. [45]) and feeding rates [37–39]. In these cases, the use of different call types signals to conspecifics where to find high-quality food [37–39]. For example, humpback whales use acoustic cues to initiate, coordinate and potentially aggregate prey when creating bubble nets around herring schools in Alaskan waters [10,19]. While minke whales are generally thought to be solitary, they are known to form larger groups (up to 40 individuals) that anecdotally include group foraging [32]. In our dataset, 14 of the 16 tags were deployed on minke whales in groups of two or more animals, with the largest group we encountered being six individuals. Our combined video and audio data confirm that Antarctic minke whales not only forage with conspecifics but that there are specific call types (*growls* and *rumbles*) associated with group feeding. While our results identify a relationship between certain call types and foraging, further examination of video data may reveal the extent to which group feeding is coordinated in this species (e.g. animals feed in synchrony or display different roles) and facilitated by the exchange of signals. Additionally, an increased number of tag deployments in conjunction with prey sampling would be valuable to understand if and how minke whales use acoustic signals to regulate fission/fusion social dynamics in response to available resources.

Interestingly, we found no evidence of the *bio-duck* sound that has been reliably attributed to this species broadly in the Southern Ocean [26]. Previous accounts have noted that this sound type is the most common call in the Western Antarctic Region during the austral winter, and is mainly absent during the summer months [28–30]. Our short recording time window during the austral summer may help to explain why no *bio-duck* calls were detected. This finding also confirms prior reports that this call exhibits strong seasonality and may possibly function as a reproductive call, although this warrants further investigation.

The scope of our study has several limitations. While the video recordings collected by our tags are exceptionally novel, they are restricted in duration relative to our acoustic recordings. Furthermore, video recordings were only collected during the day when whales were at a depth with enough light to initiate recording. Thus, there were probably times and contexts where calls were associated with the visibility or non-visibility of close conspecifics that we were unable to characterize. Additionally, our assessment of conspecifics being present based on visual confirmation within the immediate field of view of the camera is not comprehensive. While this method does limit false positives, the acoustic environment in which animals communicate is certainly beyond the visual scope of what the video cameras can see. Finally, while we have demonstrated a correlation between specific call types and two identified behavioural states, we advocate future studies combine both descriptive and experimental methods (i.e. playback experiments) to establish an understanding of the functional significance of these calls to listeners.

One of the most common tools for evaluating seasonal patterns of distribution, large-scale movement patterns and estimates of abundance of sensitive species is passive acoustic monitoring (PAM). However, the most substantial knowledge gap for PAM is the limited availability of comprehensive, expert-verified descriptions of the vocal behaviour of the species of interest [46]. The calls presented in our study improve knowledge of the acoustic repertoire of Antarctic minke whales and enable the advancement of current methods used for animal detection and classification (e.g. unsupervised feature extraction). Using these newly described sound types, we encourage others to retrospectively investigate year-round PAM datasets to assess the small and large-scale distribution patterns, migratory behaviour, and habitat use of Antarctic minke whales (as in [29]). This information may then be related to current and modelled sea-ice conditions to better predict the impacts of climatic changes on this species within a rapidly changing Antarctic region.

## 5. Conclusion

Our study provides new information on the breadth of and behavioural associations among Antarctic minke whale acoustic call types and provides an initial assessment of the behavioural and social context associated with vocal production. This information is crucial to unravelling the behaviour and ecology of this poorly known species and can be used to evaluate previously recorded passive acoustic data from the region to better characterize their distribution, abundance, and behaviour over a greater spatial and temporal extent of their range. This work offers a foundation from which to better understand the impacts of climate driven changes that affect the preferred habitat for this ice-affiliated krill predator.

## Data Availability

All biologging sensor and acoustic data are available on UCSC servers and Dryad at: https://doi.org/10.7291/D1RH5H [47]. Data are also available in the electronic supplementary material [48].
